# Serum Leptin Levels in Pemphigus: A Case Control Study

**DOI:** 10.1155/2014/853705

**Published:** 2014-05-12

**Authors:** Nikoo Mozafari, Reza M. Robati, Shima Younespour

**Affiliations:** Skin Research Center, Department of Dermatology, Shahid Beheshti University of Medical Sciences, Shohada-e-Tajrish Hospital, Shahrdari Street, Tehran 1989934148, Iran

## Abstract

*Background*. Pemphigus is an autoimmune blistering disease mediated by autoantibodies directed against keratinocyte adhesion molecules. Leptin, an adipocyte-derived hormone, plays a role in immune responses and promotes autoimmunity. *Objectives*. This study was conducted to determine whether serum leptin levels are altered in pemphigus patients and whether there is any correlation between leptin levels and the severity of disease. *Methods*. This study included 47 newly diagnosed patients with pemphigus and 43 age- and sex-matched healthy controls. Clinical characteristics and pemphigus area and activity score (PAAS) were assessed. Serum leptin levels (ng/mL) were measured by a commercial enzyme-linked immunosorbent assay (ELISA). *Results*. Patients did not differ significantly in serum leptin levels from healthy controls (median (range): 10.8 (0.1–110) ng/mL versus 12 (0.5–69.9) ng/mL and *P* = 0.53). There was no significant association between serum leptin concentrations and severity of disease measured by PAAS (*r*_*s*_ = 0.06, *P* = 0.70). *Conclusion*. The results suggest that pemphigus does not have a direct influence on serum leptin levels and the pathogenesis of pemphigus seems to be not dependent on the connection with adipose tissue.

## 1. Introduction

Leptin is a peptide hormone that functions in regulating food intake and body weight. It influences the endocrine and reproductive systems and also plays a role in immune responses [1, 2]. Both T and B cells express receptors for the long isoform of leptin. Leptin stimulates the proliferation of T cells* in vitro* and protects T cells from corticosteroid induced apoptosis [1]. Some recent studies have shown elevated serum leptin levels in patients with systemic lupus erythematosus (SLE) or rheumatoid arthritis (RA) in comparison to healthy controls [3]. Although clinical significance of this elevation remains unknown leptin antagonists may represent new possibilities for the therapy of autoimmune disorders [4].

Pemphigus is a devastating autoimmune blistering disease mediated by autoantibodies directed against keratinocyte adhesion molecules, desmoglein (Dsg3 and Dsg1), which when targeted lose their cellular adhesion properties and separate from one another to form blisters in the skin and oral cavity [5]. Whilst the production of pathogenic antibodies is key to the development of pemphigus, recent evidence implies that both T cells and B cells with autoreactivity towards Dsg3 are necessary for the pathogenesis of pemphigus disease [6]. It is also suggested that T cell response to Dsgs regulates cell dependent production of pathogenic autoantibodies by B cells [7]. Therefore, we presumed that leptin might play a role in the pathogenesis of pemphigus regarding its wide range of effect on T cells.

It is of interest whether leptin can influence the development of pemphigus disease. To the best of our knowledge there is no data on serum leptin levels in patients with pemphigus. The aim of this study was to compare serum leptin levels in recently diagnosed pemphigus patients and healthy controls and to evaluate the clinical characteristics that could be associated with leptin levels.

## 2. Patients and Methods

This was a case-control study including 47 adult patients with pemphigus and 43 healthy controls. The study received approval from our Skin Research Center ethics committee. Informed consent was obtained from all participants. Patients with pemphigus were recruited from 2 reference centers (Loghman-e-Hakim and Shohada-e-Tajrish University hospitals, Tehran, Iran). All the patients were recently diagnosed cases of pemphigus. Patients under treatment with prednisolone and immunosuppressive were not included in this study. Diagnosis of pemphigus was based on the following criteria: typical clinical findings, histological features of suprabasal or subcorneal acantholysis, and intraepithelial autoantibody deposit revealed by direct immunofluorescence. Age- and sex-matched healthy controls were selected from the partner and relatives of the patients if not affected by pemphigus or other autoimmune diseases. Those who were suffering from acute or chronic diseases or were taking any kind of medication were excluded. Pregnant and lactating women were not included.

Clinical variables such as age, weight, height, body mass index (BMI), time to diagnose of pemphigus, and disease activity according to pemphigus area and severity score (PASS) were obtained [8]. In this scoring system the cutaneous score is obtained by assessment of four distinct anatomical sites: head and neck, upper extremities, lower extremities, and trunk. The extent of the affected area in each site is rated using a 6-point score. In each site, the activity score is valued by the sum of the points given to the assigned parameters. Activity related parameter is defined based on (1) number of new blisters per day (0 to 4 points), (2) Nikolsky's sign (0 to 2 points), and (3) perilesional extension of existing blisters (0 to 3 points). The final score is then calculated by multiplying area score with activity score and a weighting factor which is defined as 0.1, 0.2, 0.3, and 0.4 for head, upper extremities, trunk, and lower extremities, respectively. The total cutaneous score is ranging from 0 to 54. For the evaluation of mucosal involvement a numerical value from 0 to 3 is given to two domains (number of affected sites and severity of the lesions) to give a score ranging between 0 and 6. The sum of cutaneous and mucosal scores provided a final score, which is described between 0 and 60 [8]. The correlation between the mucosal and cutaneous severity score obtained from PASS and anti-desmoglein antibody titers based on indirect immunofluorescence has been demonstrated earlier [9].

### 2.1. Serum Leptin Levels Evaluation

Serum samples were taken after overnight fasting. The serum was stored at −80°C until analysis. Serum leptin levels were measured by a commercial enzyme-linked immunosorbent assay (ELISA) (human leptin, Mediagnost, Germany), following the manufacturer's instructions. Briefly, samples were incubated for one hour in plates coated with capture antibody. After washing, plates were then incubated for 30 minutes with a mixture of biotinylated anti-human leptin antibody and peroxidase conjugated streptavidin. Then secondary antibody was detected by addition of substrate. Absorbance was read at 450 nm. With the use of standard curves of purified recombinant molecules, quantification was performed.

### 2.2. Statistical Methods

All data analyses were performed using the statistical software JMP, version 7 (SAS Institute Inc., Cary, NC, 1989–2007). Two-sided *P* values less than 0.05 were considered statistically significant. Continuous variables are reported as mean ± SD or as medians with total and interquartile ranges (25th–75th percentiles). Categorical data are expressed as number (percentage). The normality of continuous variables was examined using the Shapiro-Wilk's *W*-test. For the continuous variables with skewed distributions, nonparametric statistical methods were applied.

In this study, the nonparametric Mann-Whitney *U*-test was used to compare the serum leptin levels of patients with pemphigus and healthy controls. Chi-square test and Fisher's exact test, wherever appropriate, were performed for data analysis. The association between serum levels of leptin and other continuous variables was assessed with Spearman's correlation test. In addition, multiple linear regression analysis with a backward-stepwise procedure was applied to determine the parameters most predictive of the serum leptin concentration. Regression diagnostics were used to detect violations in regression modelling assumptions. The residual plot against the fitted values showed an evidence of nonconstancy of error variance. Also, a departure from normality was observed in the normal probability plot of the residuals. Thus, a Box-Cox transformation on serum leptin levels was applied to remedy these departures from the model assumptions. After applying the transformation, the model was refitted and the residual plots were employed to ascertain that the regression model was appropriate for the transformed data. Furthermore, the association between serum leptin levels and clinical characteristics including age, gender, BMI, duration of disease, and PAAS of the patients with pemphigus was examined using multiple linear regression models.

## 3. Results

Forty-seven patients with pemphigus (44 with PV and three with PF) and 43 age- and sex-matched healthy controls were recruited to this study. Baseline demographics and clinical characteristics of the study groups are presented in Table 1. Patients with pemphigus had a lower weight and BMI in comparison with healthy controls (Table 1). The two groups did not differ significantly in age, height, and self-reported smoking habit (Table 1).

**Figure 1:**
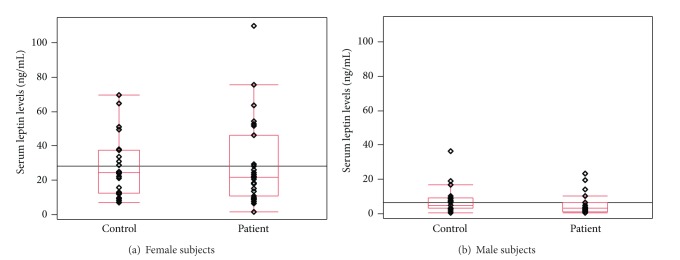
Serum leptin concentrations (ng/mL) in patients with pemphigus and healthy controls. Middle point: median; box: interquartile range (25–75 percentiles); whisker: range (excluding outliers). The width of the boxes is proportional to the number of patients contributing to the box, showing the different sample sizes in the different centers.

The median serum leptin levels were 10.8 ng/mL (IQR: 3.1–23.2 ng/mL; range: 0.1–110 ng/mL) in patients with pemphigus and 12 ng/mL (IQR: 4.9–28.7 ng/mL; range: 0.5–69.9 ng/mL) in healthy controls. The two groups did not differ significantly in the serum concentrations of leptin (*P* = 0.53). As expected, females had significantly higher median serum leptin levels than males (22.6 ng/mL (range: 1.1–110.1) for females versus 4.0 ng/mL (range: 0.1–36.3) for males, *P* < 0.0001). However, there were no significant differences between serum leptin levels of patients with pemphigus and healthy controls of either male or female subjects (Figure 1).

**Figure 2:**
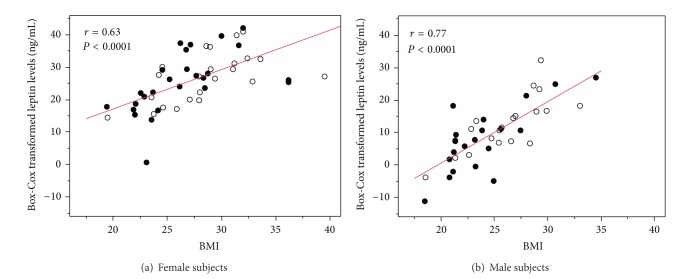
Association between Box-Cox transformed serum leptin levels and BMI in patients with pemphigus (black circles) and healthy controls (white circles) in (a) female and (b) male subjects.

In simple regression analyses, Box-Cox transformed leptin levels were correlated positively with BMI in both female and male subjects, when the two groups were included (Figure 2). In patients with pemphigus, there were significant correlations between BMI and Box-Cox transformed serum leptin concentrations (*r* = 0.66, *P* < 0.0001 in females and *r* = 0.75, *P* < 0.0001 in males). In healthy controls, significant associations were found between BMI and Box-Cox transformed leptin levels of females and males (*r* = 0.58, *P* = 0.004 in females and *r* = 0.75, *P* < 0.0001 in males).

In the multiple regression analysis, the presence of pemphigus was not associated with the serum leptin concentrations, even after adjusting for BMI and gender of subjects (*P* = 0.88). In the final model, BMI and gender of participants were significant predictors of the Box-Cox transformed leptin levels (both *P* < 0.0001). The regression model which included these two factors had the sample multiple correlation coefficient of 0.84, indicating that approximately 70% of the variance of the transformed leptin level is explained by the model.

In the patients with pemphigus, serum leptin concentrations were not associated with age (*r*_*s*_ = −0.08, *P* = 0.58), height (*r*_*s*_ = −0.22, *P* = 0.14), time until diagnosis (*r*_*s*_ = 0.24, *P* = 0.10), body surface area affected (*r*_*s*_ = 0.05, *P* = 0.73), PAAS-cutaneous score (*r*_*s*_ = 0.05, *P* = 0.73), PAAS-mucus membrane score (*r*_*s*_ = −0.02, *P* = 0.87), or PAAS-total score (*r*_*s*_ = 0.06, *P* = 0.70). Furthermore, there were no associations between serum leptin levels and clinical characteristics of patients in both female and male groups.

## 4. Discussion

In the present study, no statistically significant differences were found between median serum leptin levels of the two groups. Also there was no significant correlation between serum leptin levels and disease severity measured by PAAS. According to relatively high prevalence of pemphigus disease in our country, one of the strength points of our study was the large sample size that included 47 patients of pemphigus in this essay. All the patients were newly diagnosed and were not taking corticosteroid or other immunosuppressive drugs at the time of serum leptin levels assessment.

Consistent with report in literatures, in our study, leptin levels were significantly higher in women than in men and correlated positively with BMI [10]. Considering that factors such as gender and BMI could influence the leptin levels, female and male were evaluated separately and effect of BMI was controlled by statistical models. The BMI of the patients with pemphigus was significantly lower than that of the control volunteer. Changes in body weight may reflect poor oral intake and transcutaneous nutrient loss in patients due to oral and cutaneous erosions, respectively [11].

Some studies showed lower rate of smoking in pemphigus patients compared to normal population and this might suggest the beneficial effect of smoking in pemphigus [12]. In our study, the rate of smoking in patients (15%) was similar to previous studies but in controls (2.3%) was lower than expected [13]. However, there was not a significant difference in smoking habits between patients and controls with a borderline *P* value of 0.06. Social desirability bias may influence self-report of smoking status among control groups rather than patients. Fear of disapproval regarding disclosure of smoking status leads to misreport among controls while patients give more accurate information as they think it would be used in their disease management.

Leptin, an adipocyte-derived hormone, not only regulates food intake and energy balance but also has an important role in regulating neuroendocrine and immune functions [2, 14]. It shares structural and functional similarity with cytokines of the interleukin-6 family. During acute inflammation, circulating leptin potentiates cytokines release from monocytes and macrophages. In subjects with genetic leptin deficiency, low concentration of serum leptin contributes to increased susceptibility to infections [2]. In addition, leptin stimulates T cell mediated immunity and induces the proliferation and differentiation of hematopoietic cells. In animal experiments leptin deficient mice are resistant to the induction of several experimentally induced autoimmune diseases [15].

From clinical point of view, serum leptin levels have been evaluated in several autoimmune conditions. Elevated levels of circulating leptin and synovial leptin were reported in rheumatoid arteritis patients compared to controls or osteoarthritis patients [16]. Some authors also described elevated serum leptin levels in their patients with systemic lupus erythematosus or psoriasis in comparison with healthy controls [17–19]. However, we found no statistically significant differences between leptin levels in patients with pemphigus and the control group. It is worth noting that former diseases are T-helper-1 mediated autoimmune conditions, while pemphigus vulgaris is suggested to be a T-helper-2 type autoimmune disorder where autoreactive T-helper-2 cells to Dsg3 render B cells to secret Dsg3-specific antibodies [6, 7]. Leptin effect on autoimmune disease is mediated by a switch from a TH2 to TH1 pattern of cytokine release and consequent promotion of TH1 immune response and suppression of TH2 cell responses [20].

## 5. Conclusion

The results of our study suggest that pemphigus does not have a direct influence on leptin serum levels and it seems that the pathogenesis of pemphigus is not dependent on the connection with adipose tissue or leptin as an adipocyte-derived mediator. To the best of our knowledge, this essay is unique in the literature that assesses the leptin level in pemphigus patients and due to the considerable sample size of this uncommon autoimmune disease in this study, the outcome of this essay could be considered more definitely.

## Figures and Tables

**Table 1:** Baseline demographics and clinical characteristics of patients with pemphigus and healthy controls.

Characteristic	Patients with pemphigus (*n* = 47)	Healthy controls (*n* = 43)	*P* value
Gender			0.70
Female	27 (57.4%)	23 (53.5%)	
Male	20 (42.6%)	20 (46.5%)	
Age, years			0.69
Mean ± SD	46.81 ± 13.76	47.95 ± 13.20	
Median (range)	47 (18–82)	47 (20–80)	
Height			0.83
Mean ± SD	162.35 ± 7.36	162.76 ± 10.36	
Median (range)	162 (150–178)	161 (145–185)	
Weight			0.03
Mean ± SD	67.58 ± 12.34	72.58 ± 13.00	
Median (range)	65.5 (50–101)	72.50 (47.5–100)	
BMI			0.02
Mean ± SD	25.68 ± 4.66	27.38 ± 4.13	
Median (range)	24.46 (18.51–39.64)	27.94 (18.56–39.55)	
Smoking habit			0.06
Yes	7 (14.9%)	1 (2.3%)	
No	40 (85.1%)	42 (97.7%)	
Time until diagnosis, month			
Median (range); IQR	4 (0.5–12); (2–7)	—	
Types of pemphigus involvement			
Mucosal	10 (21.3%)	—	
Cutaneous	9 (19.1%)	—	
Mucocutaneous	28 (59.6%)	—	
BSA, %			
Median (range); IQR	3 (0–40); (0.5–10)	—	
PAAS, median (range); IQR			
Cutaneous score	2.1 (0–22.4); (0.3–6.4)	—	
Mucus membrane score	5 (0–6); (2–6)	—	
Total score^#^	6.2 (0.3–22.4); (4.1–11)	—	

Values are numbers (%) unless otherwise noted.

BMI: body mass index (calculated as weight in kilograms divided by height in meters squared); IQR: interquartile range (25th–75th percentiles); BSA: body surface area; PAAS: pemphigus area and activity score.

^#^Total score is sum of cutaneous score and mucus membrane score.
